# *“Mycobacterium massilipolynesiensis”* sp. nov., a rapidly-growing mycobacterium of medical interest related to *Mycobacterium phlei*

**DOI:** 10.1038/srep40443

**Published:** 2017-01-11

**Authors:** M. Phelippeau, S. Asmar, D. Aboubaker Osman, M. Sassi, C. Robert, C. Michelle, D. Musso, M. Drancourt

**Affiliations:** 1 Aix-Marseille Université, URMITE, UMR CNRS 7278, IRD 198, Inserm 1095, Marseille, France; 2 Centre d’Etudes et de Recherche de Djibouti (CERD), Institut de Recherche Médicinale (IRM), Djibouti; 3 Université de Rennes 1, Laboratoire de Biochimie Pharmaceutique, InsermU835-UPRES EA 2311, Rennes, France; 4 Pôle de Recherche et de Veille sur les Maladies Infectieuses Emergentes, Institut Louis Malardé, Tahiti, Polynésie Française

## Abstract

In French Polynesia, respiratory tract clinical isolate M26, displayed unusual phenotype and contradictory phylogenetic affiliations, suggesting a hitherto unidentified rapidly-growing *Mycobacterium* species. The phenotype of strain M26 was further characterized and its genome sequenced. Strain M26 genome consists in a 5,732,017-bp circular chromosome with a G + C% of 67.54%, comprising 5,500 protein-coding genes and 52 RNA genes (including two copies of the 16 S rRNA gene). One region coding for a putative prophage was also predicted. An intriguing characteristic of strain M26’s genome is the large number of genes encoding polyketide synthases and nonribosomal peptide synthases. Phylogenomic analysis showed that strain M26’s genome is closest to the *Mycobacterium phlei* genome with a 76.6% average nucleotide identity. Comparative genomics of 33 *Mycobacterium* genomes yielded 361 genes unique to M26 strain which functional annotation revealed 84.21% of unknown function and 3.88% encoding lipid transport and metabolism; while 48.87% of genes absent in M26 strain have unknown function, 9.5% are implicated in transcription and 19% are implicated in transport and metabolism. Strain M26’s unique phenotypic and genomic characteristics indicate it is representative of a new species named “*Mycobacterium massilipolynesiensis*”. Looking for mycobacteria in remote areas allows for the discovery of new *Mycobacterium* species.

Non-tuberculous mycobacteria (NTM) are isolated from human and environmental samples all over the world^1^, including tropical areas and remote countries and territories^2^. Our recent investigation of mycobacteria in French Polynesia, a remote French territory composed of 117 islands in the South Pacific^3^, indicated that rapidly-growing mycobacteria formed the most prevalent group of NTM in human respiratory tract specimens^3^. Among such organisms, sequencing the 16 S rRNA, *rpoB* and *hsp65* genes suggested that strain M26, isolated from sputum collected in August 2011 from an 86-year-old French Polynesian man living in Taravao (17 44’ S, 149 18’ W) was representative of a new species. Strain M26 displayed 91.47% *rpoB* gene sequence similarity with *Mycobacterium rhodesiae* CIP 106806^T^ (GenBank: EU109302), 98.93% 16 S rRNA gene sequence similarity with *Mycobacterium smegmatis* ATCC 19420^T^ (GenBank: NR115233) and 95.59% *hsp65* gene sequence similarity with *Mycobacterium conceptionense* CIP 108544^T^ (GenBank: AY859678)^3^. Since strain M26 was regarded as a potentially undescribed species of the genus *Mycobacterium*, it was further characterized.

For phenotype, we observed that strain M26 exhibited yellow-orange, circular, convex, smooth colonies on Middlebrook 7H10 medium. Exposing colonies to light during incubation did not change their color. Growth occurred from 28–42 °C with optimum growth at 30–37 °C, where colonies are detected at 48-hour subculture. Growth was also observed on trypticase soy agar and egg-based Coletsos medium, and Middlebrook 7H9 broth. Strain M26 exhibited Gram-positive, red Ziehl-Neelsen stained cells, which were non-motile and measured 0.54 ± 0.1 μm wide and 3.32 ± 1.46 μm on an average (Fig. 1). The catalase, niacin and Tween 80 hydrolysis tests were negative at 37 °C. Culture was positive when adding nitrobenzoic acid or up to 2% salt in Middlebrook 7H10 solid medium. Strain M26 hydrolyzed the complex polysaccharides esculin and gelatin. Further enzyme activities included pyrazinamidase, alkaline phosphatase, alpha-glucosidase, beta-glucosidase, urease but it was negative for nitrate reductase, pyrolidonyl arylamidase, beta-glucuronidase, beta-galactosidase, and N-acetyl beta-glucosaminidase. This phenotypic profile was unique, and specifically M26 strain phenotypically differed from *M. smegmatis* and *M. conceptionense*, the two closest organisms based on 16 S rRNA and *hsp*65 gene sequence similarity (Fig. 2). In particular, results of catalase, growth at 5% NaCl, Tween 80 hydrolysis, nitrate reductase, gelatin hydrolysis distinguish strain M26 from *M. smegmatis*^4^. Also, the absence of scotochromogenic colonies, negative catalase activity and negative Tween hydrolysis at 37 °C all distinguished strain M26 from *M. rhodesiae*, the closest species by *rpo*b sequencing^4^ (Fig. 2). Accordingly, M26 strain exhibited a reproducible matrix-assisted laser desorption ionization-time of flight-mass spectrometry (MALDI-TOF-MS) profile that did not match any of the profiles entered in the Bruker database (version December, 2015). Furthermore, strain M26 was susceptible to rifabutin (minimal inhibitory concentration (MIC) < 0.015 mg/L), rifampicin (MIC, 0.06 mg/L), ethambutol (MIC, 1 mg/L), clarithromycin (MIC, 2 mg/L), azithromycin (MIC, 2 mg/L), linezolid (MIC, 0.5 mg/L), amikacin (MIC, 0.25 mg/L), ciprofloxacin (MIC, 0.125 mg/L), levofloxacin (MIC, 0.06 mg/L), moxifloxacin (MIC, <0.03 mg/L), sulfamethoxazole (MIC, 1 mg/L) and resistant to penicillin G (MIC, 256 mg/L), imipenem (MIC, 64 mg/L), doxycyclin (MIC, 4 mg/L) and minocyclin (MIC, 1 mg/L). This antibiotic susceptibility profile also distinguished strain M26 from *M. rhodesiae*, which is resistant to rifampicin with a MIC of 25 mg/L. Phenotypic traits therefore suggested that strain M26 was a representative strain of a previously undescribed *Mycobacterium* species.

Further, we found that the genome of strain M26 comprised one 5,732,017-bp long chromosome without plasmid and a 67.54% GC content (Fig. 3). The genome of strain M26, comprising 12 contigs assembled into nine scaffolds, was ordered using Mauve software and *Mycobacterium gilvum* genome as nearest sequenced complete genome reference. The ordered scaffolds were then assembled into one complete sequence. Analyzing the replication origin in strain M26 genome using Ori-Finder^5^,^6^ predicted two OriC regions splited by the *dnaA* gene and located at scaffold00008 (508 and 368 bp) (Supplementary File 1). The 368 bp predicted OriC region showed homology sequence with *Mycobacterium rhodosiae* NBB3 and *Mycobacterium avium* 104 in DoriC database^7^ (Supplementary File 1). The genome of strain M26 encodes 5,500 protein-coding genes and 52 RNAs including two complete rRNA operons and 46 tRNA. A total of 4,086 genes (74.29%) were assigned as putative function (by COGs or by NR blast), whereas 166 genes (3.02%) were identified as ORFans. The remaining genes were annotated as hypothetical proteins (989 genes, 18%). A total of 2,876 proteins were found to be associated with mobilome. Eleven genes encode for bacteriocins and 100 proteins are associated with toxin/antitoxin system. We detected no gene encoding for proteins potentially involved in the resistome in the genome of strain M26. One protein was found to belong to the FxsA bacterial family of cytoplasmic membrane proteins. The molecular function of FxsA is unknown, but its over-expression in *Escherichia coli* has been shown to lessen the exclusion of phage T7 in those cells with an F plasmid. Analyzing the mobilome of strain M26 predicted one incomplete 23-kbp prophage region related to the *Herpesviridae* family, which lost the structural and replication protein required for a phage (Fig. 3, Fig. 4). We detected no gene for clustered regularly interspaced short palindromic repeats (CRISPR). We identified a large number of genes assigned to COG functional categories for transport and metabolism of lipids (8.9%), secondary metabolites biosynthesis, transport and catabolism (7.3%), amino acid transport and metabolism (6.3%) and energy production and conversion (6%) in the strain M26 genome (Table 1). This genomic content probably reflects the high lipid content in strain M26, as is known for the genus *Mycobacterium.* Mycobacterial lipids play important roles in virulence and drug susceptibility^8^. The genome of M26 also has the potential to produce secondary metabolites, with 73 genes found to be associated with polyketide synthases (PKSs) and non-ribosomal peptide synthetase (NRPSs). Some of these genes are orthologous to some *Mycobacterium tuberculosis* genes such as *Pks 13*^9^. NRPSs encode for peptide synthetase orthologous to those of *Myxococcus stipitatus* and siderophore biosynthesis protein, similar to those of *Streptomyces davawensis* and *Streptomyces griseus*. Siderophores are implicated in the acquisition of ferric iron in most microorganisms.

Phylogenomic analysis showed that the strain M26 genome is closest to *Mycobacterium phlei* genome, with average nucleotide identity of 76.6% (Fig. 5). The strain M26 genome is smaller than those of *M. smegmatis* str. mc2 155 and *M. rhodesiae* NBB3 (6.99 Mb and 6.42 Mb, respectively); its GC% content is higher than those of *M. smegmatis* str. mc2 155 and *M. rhodesiae* NBB3 (67.4% and 65.49%, respectively). We further analyzed 32 *Mycobacterium* species genomes in addition to the M26 strain genome to construct the *Mycobacterium* pangenome (Table 2). These 33 *Mycobacterium* species yielded a pangenome of 181,387 genes. The core genome (the set of genes shared by all the studied species) is composed of 1,474 orthologous genes (45.29%) and the accessory genome (the set of genes present in some but not all the species) is composed of 8,406 orthologous genes and 8,963 unique genes (genes unique to individual species) (54.71%). M26 strain encodes 361 unique genes (0.19%). A total of 14,356 genes encoded in other *Mycobacterium* species, are absent in the M26 genome (7.54%) (Fig. 6A). Functional annotation of M26 strain unique genes revealed that 84.21% have unknown function and 3.88% are implicated in lipid transport and metabolism; while 48.87% of genes absent in the M26 strain genome have unknown function, 9.5% are implicated in transcription and 19% are implicated in transport and metabolism (Fig. 6B). *M. phlei* is an environmental organism seldom responsible for opportunistic infection^10^,^11^,^12^,^13^. In a few patients, *M. phlei* was isolated from the respiratory tract, as was the case for strain M26^14^,^15^. In the situation where a clinical non-tuberculous clinical strain M26 exhibited an uncertain phylogenetic position, whole genome sequencing analysis unambiguously confirmed that it was a representative of a previously undescribed new species most closely related to *M. phlei*, with a 76.6% Average Nucleotide Identity (ANI).

The unique phenotypic and genetic characteristics of strain M26 support the fact that it is representative of a hitherto undescribed species in the genus *Mycobacterium*. Strain M26^T^ (=CSUR P1385^T^ = DSM 46850^T^) is the type strain of “*Mycobacterium massilipolynesiensis*” sp. nov. [masilipɔlinezjɛnsis], whose name is composed of *Massilia*, the Latin name for Marseilles, France, and Polynesia, referring to French Polynesia, where it was isolated. It is proposed to be a novel species belonging to the *Mycobacterium* genus, based upon *rpoB* discrimination within the nontuberculous rapidly-growing mycobacteria group and its genome features. A remarkable genomic *Mycobacterium massilipolynesiensis* feature is the large number of genes assigned to the transport and metabolism of lipids and secondary metabolite biosynthesis, transport and catabolism. Older suggestion that a new bacterial species be reported on the basis of several isolates^16^,^17^ would have prevented to draw attention of microbiologists to this new species. Alternatively, its free availability in public culture collection will allow microbiologists to report on additional isolates in order to precise the potential of *M. massilipolynesiensis* as an opportunistic pathogen; on the wave of culturomics hundreds of new bacterial species with only one cultured representative^18^,^19^.

## Methods

To further characterize strain M26, growth was observed after sub-culture on Middlebrook 7H10 solid agar (Becton Dickinson, Le Pont de Claix, France) at 28–42 °C for two weeks. The development of colonies was inspected daily by the naked eye. Optical microscopy was performed using a Leica DM 2500 microscope (Leica, Wetzlar, Germany) after Gram staining and Ziehl-Neelsen staining and pictures were taken using a Nikon Digital Sight DS*-*U1 Camera System (Nikon, Tokyo, Japan). Transmission electron microscopy (Morgani 268D; Philips, Eindhoven, The Netherlands) was used to determine the size of the microorganisms after negative staining. Carbon source utilization and enzyme activities were determined by inoculating a API^®^ Coryne strip (bioMérieux) as indicated by the manufacturer. For Tween 80 hydrolysis, two drops of Tween 80 reagent (Sigma-Aldrich, L’Isle-d’Abeau, France) and neutral red as indicator were mixed with 1 mL of sterile distilled water into a screw-cap tube. The tube was inoculated with a loopful of the microorganism and incubated at 37 °C in the dark with the cap tight. The tube was visually inspected daily up for to 10 days of incubation. Niacin production was detected using BBL ™Taxo ™TB Niacin test strips (Becton Dickinson) as described by the manufacturer. Salt tolerance of strain M26 was tested by supplementing the Middlebrook 7H10 solid medium with 0–5% NaCl. Matrix-assisted laser-desorption/ionization time-of-flight mass spectrometry (MALDI-TOF- MS) protein analysis was carried out as previously described^20^. Drug susceptibility was tested using the broth microdilution method in Middlebrook 7H9 medium (Becton Dickinson) as previously described^21^.

### Genome sequencing and assembly

DNA was extracted from strain M26 and cultured for seven days in one MGIT medium (Becton Dickinson) tube supplemented with 0.8 mL of Oleic Albumin Dextrose Catalase (OADC) enrichment (Becton Dickinson) at 37 °C. The inoculated broth was then centrifuged at 8,000 g for 10 min and the pellet was resuspended in 160 μL of G2 buffer (EZ1 DNA Tissue kit, Qiagen, Courtaboeuf, France), mixed with 40 μL of lysozyme at 25 mg/mL (Euromedex, Strasbourg, France). DNA was extracted using a BioRobot EZ1 Advanced XL (Qiagen). Genomic M26 DNA was sequenced on the MiSeq Technology (Illumina Inc, San Diego, CA, USA) with both paired-end and mate-pair applications. The gDNA was quantified by a Qubit assay with the high sensitivity kit (Life Technologies, Carlsbad, CA, USA) to 95.1 ng/μL and was barcoded respectively in order to be mixed with 12 other projects with the Nextera Mate Pair sample prep kit (Illumina) and with 15 others projects with the Nextera XT DNA sample prep kit (Illumina). To prepare the paired-end library, dilution was performed to require 1ng of each genome as input. The “tagmentation” step fragmented and tagged the DNA. Limited cycle PCR amplification (12 cycles) completed the tag adapters and introduced dual-index barcodes. After purification on AMPure XP beads (Beckman Coulter Inc, Fullerton, CA, USA), the libraries were normalized on specific beads according to the Nextera XT protocol (Illumina). Normalized libraries were pooled for sequencing on the MiSeq. The pooled single strand library was loaded onto the reagent cartridge and then onto the instrument along with the flow cell. Automated cluster generation and paired end sequencing with dual index reads were performed in a single 39-hour run in 2 × 251-bp. Total information of 5.9 Gb was obtained from a 643 K/mm^2^ cluster density with cluster passing quality control filters of 91.8% (12,365,000 clusters). Within this run, the index representation for M26 was 6.43%. The 729,975 paired-end reads were trimmed and filtered according to the read qualities. Two separated mate-pair libraries were prepared with 1.36 and 1.5 μg of genomic DNA using the Nextera mate pair Illumina guide. The genomic DNA sample was simultaneously fragmented and tagged with a mate pair junction adapter. The fragmentation profiles were validated on an Agilent 2100 BioAnalyzer (Agilent Technologies Inc, Santa Clara, CA, USA) with a DNA 7500 labchip. The size of the DNA fragments ranged from 1 to 11 kb with an optimal size at 4.58 and 5.27 kb, respectively. No size selection was performed and 503 and 600 ng respectively of tagmented fragments were circularized. The circularized DNA was mechanically sheared to small fragments with an optimal at 871 bp on the Covaris device S2 in microtubes (Covaris, Woburn, MA, USA). The libraries profiles were quantified and visualized on a High Sensitivity Bioanalyzer LabChip (Agilent Technologies Inc). The libraries were normalized at 2 nM, pooled, denatured and diluted at 15 pM for sequencing. One M26 library was loaded in two different 2 × 251-bp runs (at 506 K/mm^2^ cluster density with cluster passing quality control filters of 97%, the index representation was determined at 5.71%, at 852 K/mm^2^ cluster density with cluster passing quality control filters of 94.53%, the index representation was 4.04%). The second library was loaded and the run performed at 587 K/mm^2^ cluster density with cluster passing quality control filters of 96.3% the index representation was 7.44%). These three runs in mate-pair strategy generated a total of 2,004,521 pass filter paired-reads for M26. Illumina reads were trimmed using Trimmomatic^22^, then assembled using Spades software^23^,^24^. Contigs were combined together by SSpace^25^ and Opera software v1.2^26^, helped by GapFiller V1.10^27^ to reduce the set. Some manual refinements using CLC Genomics v7 software (CLC bio, Aarhus, Denmark) and homemade tools made in Python improved the genome. Finally, the draft genome of strain M26 consists into nine scaffolds. The replication origin in strain M26 genome was predicted using Ori-Finder^5^,^6^ (http://tubic.tju.edu.cn/Ori-Finder/) and homology with other OriC regions was predicted using blast algorithm in DoriC database^7^ (http://tubic.tju.edu.cn/doric/). The 16 S rRNA gene sequence of strain M26 was deposited in GenBank under accession number LN909503 and its complete genome under accession number FAXD00000000.

### Genome annotation

Non-coding genes and miscellaneous features were predicted using RNAmmer^28^, ARAGORN^29^, Rfam^30^, PFAM^31^, and Infernal^32^. Coding DNA sequences (CDSs) were predicted using Prodigal^33^ and functional annotation was achieved using BLAST+^34^ and HMMER3^35^ against the UniProtKB database^36^. To refine the search of mobile genetic elements in the genome of strain M26, PHAST software predicted prophage^37^ completed by the Actinobacteriophage Database at phageDB.org and the online plasmid search tool: https://plasmid.med.harvard.edu/PLASMID/Home.xhtml. Phylogenomic tree was constructed using an identity matrix based on Mauve program genome alignment^38^. Average Nucleotide Identity values were calculated between strain M26 and sequenced *Mycobacterium* species genomes using BLASTN and a home-made software, following the algorithm described by Goris *et al*.^39^. The genomes of 32 *Mycobacterium* species were downloaded from Genbank (Table 2). The ORFeome of these species, in addition to M26 genome, were annotated using Prodigal^33^ in order to normalize ORFs predictions. The Pangenomic analyses were computed using OrthoMCL software with >60% amino acid identiy in all-against-all BLASTP algorithm^40^.

## Additional Information

**How to cite this article**: Phelippeau, M. *et al*. “*Mycobacterium massilipolynesiensis*” sp. nov., a rapidly-growing mycobacterium of medical interest related to *Mycobacterium phlei. Sci. Rep.* **7**, 40443; doi: 10.1038/srep40443 (2017).

**Publisher's note:** Springer Nature remains neutral with regard to jurisdictional claims in published maps and institutional affiliations.

## Supplementary Material

Supplementary Information

## Figures and Tables

**Figure 1:**
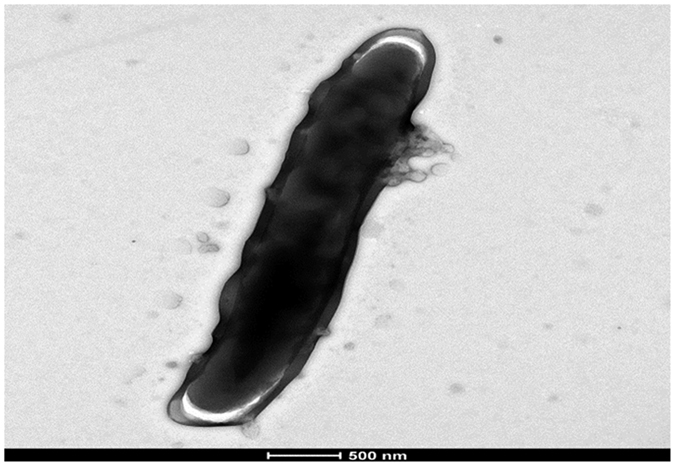
Transmission electron microscopy of “*Mycobacterium massilipolynesiensis*” sp. nov. strain M26^T^, using a Morgani 268D (Philips) at an operating voltage of 60 kV. The scale bar represents 500 nm.

**Figure 2:**
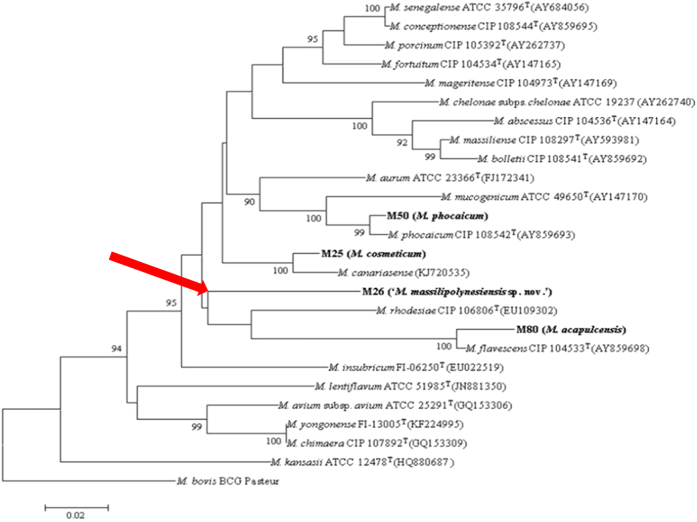
Phylogenetic tree based on *rpoB* sequences, bootstrapped 100 times, highlighting the position of *Mycobacterium massilipolynesisensis* sp. nov. M26^T^ (in bold) relative to other nontuberculous mycobacteria (branch point highlighted by a red arrow). The tree was constructed using the ClustalW and MEGA5 software programs. Only homology distance values above 90% are displayed in the tree. The corresponding GenBank accession numbers are displayed in parentheses. Bar: 0.02 substitution per nucleotide position.

**Figure 3:**
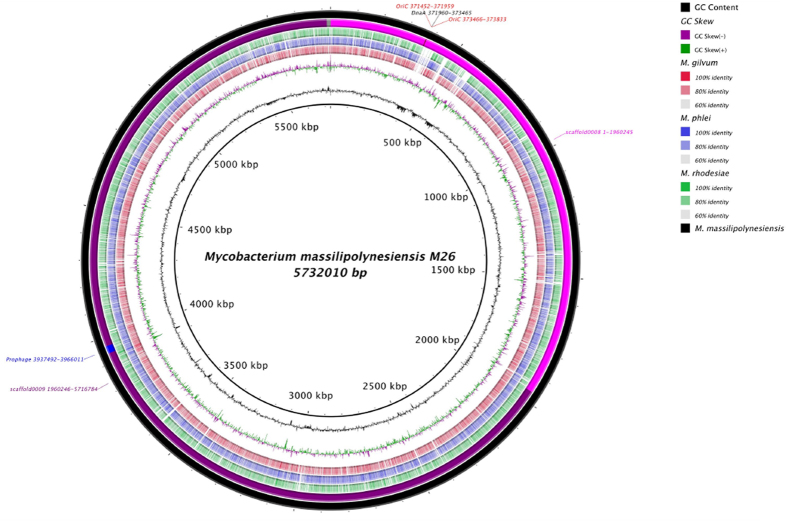
Graphical circular map of M26 genome. Figure 3 shows the genome sequence of M26 strain (Black) compared to three *Mycobacterium* genomes. The outermost ring highlights the genome sequence of M26 and annotated Prophage and OriC regions, shown in blue and red respectively. The scaffolds were ordered using Mauve software and the complete genome sequence of *M. gilvum* (Second circle). The remaining rings show BLAST comparison with *M. gilvum. M. phlei* and *M. rhodesiae*. The innermost rings show GC skew (purple/green) and GC content (black).

**Figure 4:**
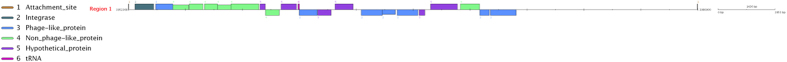
Genomic organization of M26 uncomplete prophage region. The brown color represents the attachment site; the green the integrase, the blue indicates prophage-like proteins.

**Figure 5:**
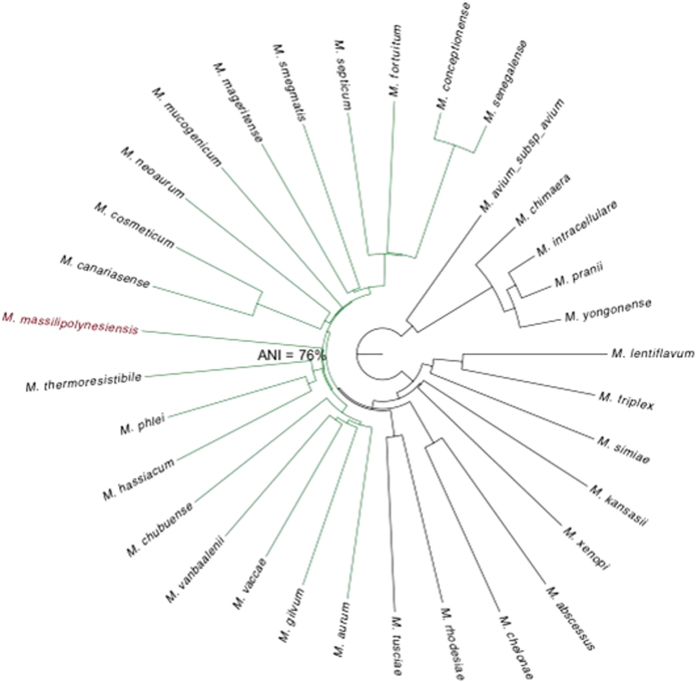
Aligned whole genomes phylogenomic tree based on Mauve alignment identity matrix. ANI, Average Nucleotide Identity.

**Figure 6:**
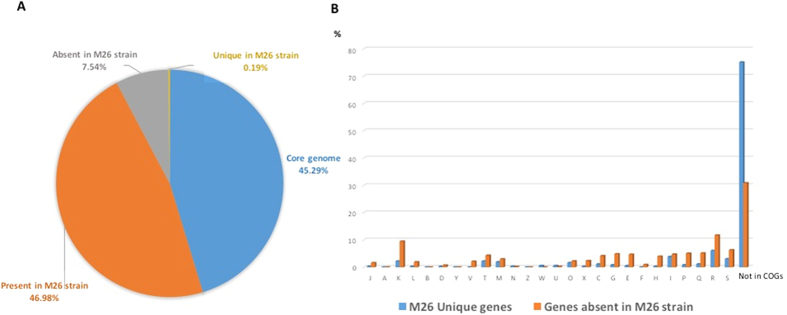
Pangenomic analysis and functional annotation of 33 *Mycobacterium* genome species. (**A**) Pangenome distribution (**B**) COGs distribution of unique genes and genes absent in M26 strain genome.

**Table 1:** Number of genes associated with the 25 general COG functional categories.

Code	Value	% of total	Description
[J]	157	2.8545454	Translation
[A]	1	0.018181818	RNA processing and modification
[K]	303	5.509091	Transcription
[L]	125	2.2727273	Replication, recombination and repair
[B]	0	0	Chromatin structure and dynamics
[D]	27	0.49090907	Cell cycle control, mitosis and meiosis
[Y]	0	0	Nuclear structure
[V]	39	0.7090909	Defense mechanisms
[T]	109	1.9818181	Signal transduction mechanisms
[M]	133	2.418182	Cell wall/membrane biogenesis
[N]	11	0.2	Cell motility
[Z]	0	0	Cytoskeleton
[W]	0	0	Extracellular structures
[U]	26	0.47272727	Intracellular trafficking and secretion
[O]	104	1.8909091	Posttranslational modification, protein turn-over, chaperones
[C]	328	5.9636364	Energy production and conversion
[G]	199	3.618182	Carbohydrate transport and metabolism
[E]	346	6.290909	Amino acid transport and metabolism
[F]	77	1.4000001	Nucleotide transport and metabolism
[H]	131	2.3818183	Coenzyme transport and metabolism
[I]	487	8.854546	Lipid transport and metabolism
[P]	215	3.909091	Inorganic ion transport and metabolism
[Q]	399	7.254545	Secondary metabolites biosynthesis, transport and catabolism
[R]	647	11.763636	General function prediction only
[S]	259	4.709091	Function unknown
_	2091	38.01818	Not in COGs

The % total is based on the total number of protein coding genes in the annotated genome.

**Table 2:** Mycobacterial genomes used in pangenomic analysis.

Species	Genbank accesion number	No. of ortthologous genes	No. of unique genes
*Mycobacterium abscessus*	NC_010397.1	4615	340
*Mycobacterium aurum*	CVQQ01000001.1	5519	392
*Mycobacterium avium subsp. avium*	NZ_ACFI01000001.1	4440	144
*Mycobacterium canariasense*	BCSY01000001.1	6204	591
*Mycobacterium chelonae*	NZ_CP010946.1	4445	329
*Mycobacterium chimaera*	LJHN01000001.1	5805	269
*Mycobacterium chubuense*	CP003053	5024	213
*Mycobacterium conceptionense*	LFOD01000001.1	6498	531
*Mycobacterium cosmeticum*	CCBB010000001.1	6117	151
*Mycobacterium fortuitum*	NZ_CP011269.1	5800	193
*Mycobacterium gilvum*	NC_009338.1	5219	140
*Mycobacterium hassiacum*	NZ_KB903840.1	4587	248
*Mycobacterium indicus pranii*	NC_018612.1	5117	62
*Mycobacterium intracellulare*	NC_016946.1	4964	65
*Mycobacterium kansasii*	CP006835.1	5059	556
*Mycobacterium lentiflavum*	CTEE01000001.1	6026	329
*Mycobacterium mageritense*	CCBF010000001.1	7127	549
*Mycobacterium massilipolynesiensis*	FAXD000000001.1	5125	361
*Mycobacterium mucogenicum*	CYSI01000001.1	5497	495
*Mycobacterium neoaurum*	CP006936.2	4878	225
*Mycobacterium phlei*	NZ_AJFJ01000001.1	5281	316
*Mycobacterium rhodesiae*	NC_016604.1	5977	292
*Mycobacterium senegalense*	LDPU01000001.1	6413	261
*Mycobacterium septicum*	CBMO010000001.1	6334	258
*Mycobacterium simiae*	CBMJ020000001.1	5162	320
*Mycobacterium smegmatis*	NC_008596.1	6417	260
*Mycobacterium thermoresistibile*	NZ_AGVE01000001.1	4381	215
*Mycobacterium triplex*	CCAU010000001.1	5787	203
*Mycobacterium tusciae*	NZ_AGJJ01000001.1	6422	571
*Mycobacterium vaccae*	NZ_JH814683.1	5666	251
*Mycobacterium vanbaalenii*	NC_008726.1	5866	292
*Mycobacterium xenopi*	NZ_AJFI01000001.1	3994	293
*Mycobacterium yongonense*	NC_021715.1	5020	86

## References

[b1] Menzies D. & Nahid P. Update in tuberculosis and nontuberculous mycobacterial disease 2012. Am. J. Respir. Crit. Care Med. 188, 923–927 (2013). 24127799 10.1164/rccm.201304-0687UP

[b2] Lillis J. V. & Ansdell D. Outbreak of nontuberculous mycobacterial disease in the central Pacific. Dermatol. Clin. 29, 9–13 (2011). 21095522 10.1016/j.det.2010.09.008

[b3] Phelippeau M., Osman D. A., Musso D. & Drancourt M. Epidemiology of nontuberculous mycobacteria in French Polynesia. J. Clin. Microbiol. 53, 3798–804 (2015). 26400787 10.1128/JCM.01560-15 PMC4652114

[b4] Gordon R. E. & Smith M. M. Rapidly growing, acid fast bacteria. I. Species’ descriptions of *Mycobacterium phlei* Lehmann and Neumann and *Mycobacterium smegmatis* (Trevisan) Lehmann and Neumann. J. Bacteriol. 66, 41–8 (1953). 13069464 10.1128/jb.66.1.41-48.1953 PMC357089

[b5] Tsukamura M., Mizuno S. & Tsukamura S. Numerical analysis of rapidly, scotochromogenic mycobacteria including *Mycobacteruium obuense* sp. nov., nom. rev., *Mycobacteruium rhodesiae* sp. nov., nom rev., *Mycobacteruium aichiense* sp. nov., nom. rev., *Mycobacteruium chubuense* sp. nov., nom. rev., *Mycobacteruium tokaiense* sp. nov., nom. rev Inter. J. Syst. Bact. 31, 263–275 (1981).

[b6] Gao F. & Zhang C. T. Ori-Finder: a web-based system for finding oriCs in unannotated bacterial genomes. BMC Bioinformatics. 9, 79 (2008). 18237442 10.1186/1471-2105-9-79 PMC2275245

[b7] Gao F., Luo H. & Zhang C. T. DoriC 5.0: an updated database of oriC regions in both bacterial and archaeal genomes. Nucl Acids Res. 41, 90–93 (2013). 23093601 10.1093/nar/gks990 PMC3531139

[b8] Ritu B. M. & Hiroshi N. Mycobacterial outer membrane is a lipid bilayer and the inner membrane is unusually rich in diacyl phosphatidylinositol dimannodides. Proc. Natl. Acad. Sci. USA 111, 4958- 4963 (2014). 24639491 10.1073/pnas.1403078111 PMC3977252

[b9] Portevin D. . A polyketide synthase catalyzes the last condensation step of mycolic acid biosynthesis in mycobacteria and related organisms. Proc. Natl. Acad. Sci. USA 101, 314–319 (2004) 14695899 10.1073/pnas.0305439101 PMC314182

[b10] Aguilar J. L., Sanchez E. E., Carrillo C., Alarcón G. S. & Silicani A. Septic arthritis due to *Mycobacterium phlei* presenting as infantile Reiter’s syndrome. J. Rheumatol. 16, 1377–1378 (1989). 2810265

[b11] Spiegl P. V. & Feiner C. M. *Mycobacterium phlei* infection of the foot: a case report. Foot Ankle Int. 15, 680–683 (1994). 7894643 10.1177/107110079401501211

[b12] Paul E. & Devarajan P. *Mycobacterium phlei* peritonitis: a rare complication of chronic peritoneal dialysis. Pediatr. Nephrol. 12, 67–68 (1998). 9502573 10.1007/s004670050407

[b13] Karnam S. . *Mycobacterium phlei,* a previously unreported cause of pacemaker infection: thinking outside the box in cardiac device infections. Cardiol. J. 18, 687–690 (2011). 22113758 10.5603/cj.2011.0034

[b14] Lima C. A. . Nontuberculous mycobacteria in respiratory samples from patients with pulmonary tuberculosis in the state of Rondônia, Brazil. Mem. Inst. Oswaldo Cruz. 108, 457–462 (2013). 23827995 10.1590/0074-0276108042013010 PMC3970618

[b15] Hsiao C. H., Lin Y. T., Lai C. C. & Hsueh P. R. Clinicopathologic characteristics of nontuberculous mycobacterial lung disease in Taiwan. Diagn. Microbiol. Infect. Dis. 68, 228–235 (2010). 20846814 10.1016/j.diagmicrobio.2010.06.008

[b16] Christensen H. . Is characterization of a single isolate sufficient for valid publication of a new genus or species? Proposal to modify recommendation 30b of the Bacteriological Code (1990 Revision). Int. J. Syst. Evol. Microbiol. 51, 2221–2225 (2001). 11760965 10.1099/00207713-51-6-2221

[b17] Janda J. M. & Abbott S. L. Bacterial identification for publication: when is enough enough? J. Clin. Microbiol. 40, 1887–1891 (2002). 12037039 10.1128/JCM.40.6.1887-1891.2002 PMC130684

[b18] Lagier J. C. . The rebirth of culture in microbiology through the example of culturomics to study human gut microbiota. Clin. Microbiol. Rev. 28, 237–264 (2015). 25567229 10.1128/CMR.00014-14 PMC4284300

[b19] Lagier J. C. . Culture of previously uncultured members of the human gut microbiota by culturomics. Nature Microbiol. (2016 In press). 10.1038/nmicrobiol.2016.203 PMC12094094 27819657

[b20] El Khéchine A., Couderc C., Flaudrops C., Raoult D. & Drancourt M. Matrix-assisted laser desorption/ionization time-of-flight mass spectrometry identification of mycobacteria in routine clinical practice. PLoS ONE 6, e24720 (2011). 21935444 10.1371/journal.pone.0024720 PMC3172293

[b21] Brown-Elliott B. A., Nash K. A. & Wallace R. J. Jr. Antimicrobial susceptibility testing, drug resistance mechanisms, and therapy of infections with nontuberculous mycobacteria. Clin. Microbiol. Rev. 25, 545–582 (2012). 22763637 10.1128/CMR.05030-11 PMC3416486

[b22] Lohse M. . RobiNA: a user-friendly, integrated software solution for RNA-Seq-based transcriptomics. Nucleic Acids Res. 40 (Web Server issue), W622–627 (2012). 22684630 10.1093/nar/gks540 PMC3394330

[b23] Nurk S. . Assembling single-cell genomes and mini-metagenomes from chimeric MDA products. J. Comput. Biol. 20, 714–737 (2013). 24093227 10.1089/cmb.2013.0084 PMC3791033

[b24] Bankevich A. . SPAdes: a new genome assembly algorithm and its applications to single-cell sequencing. J. Comput. Biol. 19, 455–477 (2012). 22506599 10.1089/cmb.2012.0021 PMC3342519

[b25] Boetzer M., Henkel C. V., Jansen H. J., Butler D. & Pirovano W. Scaffolding pre-assembled contigs using SSPACE. Bioinformatics 27, 578–579 (2011). 21149342 10.1093/bioinformatics/btq683

[b26] Gao S., Sung W. K. & Nagarajan N. Opera: reconstructing optimal genomic scaffolds with high-throughput paired-end sequences. J. Comput. Biol. 18, 1681–1691 (2011). 21929371 10.1089/cmb.2011.0170 PMC3216105

[b27] Boetzer M. & Pirovano W. Toward almost closed genomes with GapFiller. Genome Biol. 13, R56 (2012). 22731987 10.1186/gb-2012-13-6-r56 PMC3446322

[b28] Lagesen K. . RNAmmer: consistent and rapid annotation of ribosomal RNA genes. Nucleic Acids Res. 35, 3100–3108 (2007). 17452365 10.1093/nar/gkm160 PMC1888812

[b29] Laslett D. & Canback B. ARAGORN, a program to detect tRNA genes and tmRNA genes in nucleotide sequences. Nucleic Acids Res. 32, 11–16 (2004). 14704338 10.1093/nar/gkh152 PMC373265

[b30] Griffiths-Jones S., Bateman A., Marshall M., Khanna A. & Eddy S. R. Rfam: an RNA family database. Nucleic Acids Res. 31, 439–441 (2003). 12520045 10.1093/nar/gkg006 PMC165453

[b31] Punta M. . The Pfam protein families database. Nucleic Acids Res. 40, D290–D301 (2012). 22127870 10.1093/nar/gkr1065 PMC3245129

[b32] Nawrocki E. P., Kolbe D. L. & Eddy S. R. Infernal 1.0: inference of RNA alignments. Bioinformatics 25, 1335–1337 (2009). 19307242 10.1093/bioinformatics/btp157 PMC2732312

[b33] Hyatt D. . Prodigal: prokaryotic gene recognition and translation initiation site identification. BMC Bioinformatics 11, 119 (2010). 20211023 10.1186/1471-2105-11-119 PMC2848648

[b34] Camacho C. . BLAST+: architecture and applications. BMC Bioinformatics 10, 421 (2009). 20003500 10.1186/1471-2105-10-421 PMC2803857

[b35] Eddy S. R. Accelerated profile HMM searches. PLoS Comp. Biol. 7, e1002195 (2011). 10.1371/journal.pcbi.1002195 PMC3197634 22039361

[b36] The UniProt Consortium. Ongoing and future developments at the Universal Protein Resource. Nucleic Acids Res. 39, D214–D219 (2011). 21051339 10.1093/nar/gkq1020 PMC3013648

[b37] Zhou Y., Liang Y., Lynch K. H., Dennis J. J. & Wishart D. S. PHAST: A Fast Phage Search Tool. Nucleic Acids Res. 39 (Web Server issue), W347–352 (2011). 21672955 10.1093/nar/gkr485 PMC3125810

[b38] Darling A. E., Mau B. & Perna N. T. ProgressiveMauve: multiple genome alignment with gene gain, loss and rearrangement. PLoS One 5, e11147 (2010). 20593022 10.1371/journal.pone.0011147 PMC2892488

[b39] Goris J. . DNA-DNA hybridization values and their relationship to whole-genome sequence similarities. Int. J. Syst. Evol. Microbiol. 57, 81–91 (2007). 17220447 10.1099/ijs.0.64483-0

[b40] Li L., Stoeckert J., Christian J. & Roos D. S. OrthoMCL: identification of ortholog groups for eukaryotic genomes. Genome Res. 13, 2178–89 (2003). 12952885 10.1101/gr.1224503 PMC403725

