# Supplementary material for: “Mycobacterium massilipolynesiensis” sp. nov., a rapidly-growing mycobacterium of medical interest related to Mycobacterium phlei
Source: Sci Rep. 2017 Jan 11;7:40443. doi: 10.1038/srep40443 (PMC5225428; doi:10.1038/srep40443)
Supplement: Supplementary Information [file srep40443-s1.pdf]

“*Mycobacterium massilipolynesiensis*” sp. nov., a rapidly-growing mycobacterium of medical interest related to *Mycobacterium phlei*

Running title: *Mycobacterium massilipolynesiensis,* new species

M. Phelippeau[1], S. Asmar[1], D. Aboubaker Osman[1, 3], M. Sassi[4], C. Robert[1], C. Michelle[1], D. Musso[2] & M. Drancourt[1#]

SUPPLEMENTARY INFORMATION

Supplementary File 1. Information of *“Mycobacterium massilipolynesiensis”* genome and oriC region.

DnaA box distribution [DnaA box distribution]
OriC length 508 nt
OriC AT content 0.4055
The number of DnaA box 5
The location of oriC region
371452..371959 nt
The location of dnaA gene 371960..373465 nt
The extremes of GC disparity 211707 nt (minimum), 1942326 nt (maximum)
The extremes of AT disparity 1930273 nt (minimum), 753915 nt (maximum)
The extremes of RY disparity 216283 nt (minimum), 1952415 nt (maximum)
The extremes of MK disparity 1929850 nt (minimum), 345919 nt (maximum)
Note Note that the E. coli perfect DnaA box (ttatccaca) was searched for with no more than two mismatches. Note that the oriC region is closely next to a dnaA gene (371960..373465 nt). Note that the oriC region is closely next to a dnaN gene (370258..371451 nt).
Z-curves [Figure1] [Figure2]
OriC Sequence The DnaA boxes identified in the below sequence are capitalized and also marked in bold, if any.
agtgtcccttcatctgcccaacaaccccgaccagcgcacacccagcgccgacggcgccag
gcgagcgtgatctgtgagcgacagccgcggaacggcagtcagaagaacaaccgtagagcg
tcggacgccatcttgaaagctaatcgatcacggtgacaaaaccgctgtgagtcacctcgc
ggcggctgtgcatgcgatcttccccagccccttctttcaaggagaaatctcagaagagat
atcggcaacagtaataggggctgtgcatcgTGTGGATCGaggagcccaacgcctgctcgg
atgtgattcggcggTGTGGATGGatTGTGGATGGctgggggcgccgggccggcccgatgt
ggaagtcgcggggttgttcatcaccatccccggttcggcgcggttgttacccgatgTTGT
CCACAgcggTGTGCACAAgagcagatgtgacgactgtgacggctgcgtccaaagtttttt
ggccgcaacgacggaatgacagggattt
The information of genome and oriC region
Genome size 1960245 nt
Genome GC content 0.6720
DnaA box distribution [DnaA box distribution]
OriC length 368 nt
OriC AT content 0.4239
The number of DnaA box 3
The location of oriC region
373466..373833 nt
The location of dnaA gene 371960..373465 nt
The extremes of GC disparity 211707 nt (minimum), 1942326 nt (maximum)
The extremes of AT disparity 1930273 nt (minimum), 753915 nt (maximum)
The extremes of RY disparity 216283 nt (minimum), 1952415 nt (maximum)
The extremes of MK disparity 1929850 nt (minimum), 345919 nt (maximum)
Note Note that the E. coli perfect DnaA box (ttatccaca) was searched for with no more than two mismatches. Note that the oriC region is closely next to a dnaA gene (371960..373465 nt).
Z-curves [Figure1] [Figure2]

OriC Sequence The DnaA boxes identified in the below sequence are capitalized and also marked in bold, if any.

cgacagatccccctggtccgaggtcgggcaacaaataaacgagacgacgacaaaCTGTCC
ACAtagTTATCCACAggTGTGGACAGgacagtcgtcctcgcctcgcagcatatcggcgga
aggtatcgggtcggcgggtaatccggtcaatcagtgttgtctggactccgaccgacgtcc
cctcccggttgtaactcccactgccgcttcgaatctgaaacggctctggcagaagctaac
agttttcccggcgggtgccaaccgttctgcaacattacgatcacgctgattgtgacccag
gtcgctctcgcgtgacagttgtgacgccagtaaatctgtgcgtcgggaggcggtactgac
aaggcggt

BLASTN 2.2.10 [Oct-19-2004]

Reference:
Altschul, Stephen F., Thomas L. Madden, Alejandro A. Schäffer,
Jinghui Zhang, Zheng Zhang, Webb Miller, and David J. Lipman (1997),
"Gapped BLAST and PSI-BLAST: a new generation of protein database search
programs", Nucleic Acids Res. 25:3389-3402.

Query=
(368 letters)

Database: oriCdb.nt
2733 sequences; 1,354,891 total letters

Searching......done

Sequences producing significant alignments:	Score (bits)	E Value
ORI95040348;Circular;Type 4;NC_016604.1;Mycobacterium rhode...	88	2e-18
ORI60040083;Circular;Type 4;NC_008595.1;Mycobacterium avium...	64	3e-11

>ORI95040348;Circular;Type 4;NC_016604.1;Mycobacterium rhodesiae NBB3 chromosome; Bacteria,
Actinobacteria, Actinobacteridae, Actinomycetales,
Corynebacterineae, Mycobacteriaceae,
Mycobacterium.;6415739 nt#0.6549; 1849907..1850471
nt;565 nt#0.3912#1;1850472..1851980 nt;1852318 nt
(minimum), 5098566 nt (maximum);-#
Length = 565

Score = 87.7 bits (44), Expect = 2e-18
Identities = 56/60 (93%)
Strand = Plus / Minus

```
Query: 217 gaaacggctctggcagaagctaacagttttcccggcgggtgccaaccgttctgcaacatt 276
           |||||||| ||||||||||||||||||||||   |||||||||||||||||||||||||||||
Sbjct: 344 gaaacggcactggcagaagctaacagttttcttcgcgggtgccaaccgttctgcaacatt 285
```

>ORI60040083;Circular;Type 4;NC_008595.1;Mycobacterium avium 104; Bacteria, Actinobacteria,
Actinobacteridae, Actinomycetales, Corynebacterineae,
Mycobacteriaceae, Mycobacterium, Mycobacterium avium
complex (MAC).;5475491 nt#0.6899; 5474887..32 nt;637
nt#0.3972#2;33..1529, 3923290..3924789 nt;16940 nt
(minimum), 2712821 nt (maximum);Note that the DnaA box
motif (tt[g/c]tccaca) was looked for with no more than
one mismatch instead of E. coli perfect DnaA box
(ttatccaca). #
Length = 637

Score = 63.9 bits (32), Expect = 3e-11
Identities = 35/36 (97%)
Strand = Plus / Minus

```
Query: 53  aactgtccacatagttatccacaggtgtggacagga 88
           |||||||||||||||||| |||||||||||||||||
Sbjct: 540 aactgtccacatagttatacacaggtgtggacagga 505
```

Database: oriCdb.nt
Posted date: Feb 27, 2013 11:38 AM
Number of letters in database: 1,354,891
Number of sequences in database: 2733

Lambda	K	H
1.37	0.711	1.31

Gapped

Lambda	K	H
1.37	0.711	1.31

Matrix: blastn matrix:1 -3
Gap Penalties: Existence: 5, Extension: 2
Number of Hits to DB: 2235
Number of Sequences: 2733
Number of extensions: 2235
Number of successful extensions: 1113
Number of sequences better than 1.0e-10: 2

Number of HSP's better than 0.0 without gapping: 2
Number of HSP's successfully gapped in prelim test: 0
Number of HSP's that attempted gapping in prelim test: 1109
Number of HSP's gapped (non-prelim): 4
length of query: 368
length of database: 1,354,891
effective HSP length: 15
effective length of query: 353
effective length of database: 1,313,896
effective search space: 463805288
effective search space used: 463805288
T: 0
A: 0
X1: 11 (21.8 bits)
X2: 15 (29.7 bits)
S1: 12 (24.3 bits)
S2: 32 (63.9 bits)
